# Use of Renin-Angiotensin System Inhibitors Is Associated with Reduction of Fracture Risk in Hemodialysis Patients

**DOI:** 10.1371/journal.pone.0122691

**Published:** 2015-04-13

**Authors:** Suguru Yamamoto, Ryo Kido, Yoshihiro Onishi, Shingo Fukuma, Tadao Akizawa, Masafumi Fukagawa, Junichiro J. Kazama, Ichiei Narita, Shunichi Fukuhara

**Affiliations:** 1 Department of Clinical Nephroscience, Niigata University Graduate School of Medical and Dental Sciences, Niigata, Japan; 2 Division of Clinical Nephrology and Rheumatology, Niigata University Graduate School of Medical and Dental Science, Niigata, Japan; 3 Institute for Health Outcomes and Process Evaluation Research (iHope International), Kyoto, Japan; 4 Medical Examination Center, Inagi Municipal Hospital, Tokyo, Japan; 5 Department of Healthcare Epidemiology, School of Public Health in the Graduate School of Medicine, Kyoto University, Kyoto, Japan; 6 Division of Nephrology, Department of Medicine, Showa University School of Medicine, Tokyo, Japan; 7 Division of Nephrology, Endocrinology and Metabolism, Tokai University School of Medicine, Isehara, Japan; 8 Blood Purification Center, Niigata University Medical and Dental Hospital, Niigata, Japan; 9 Center for Innovative Research for Communities and Clinical Excellence (CIRC^2^LE), Fukushima Medical University, Fukushima, Japan; University of Sao Paulo Medical School, BRAZIL

## Abstract

**Background:**

Patients with chronic kidney disease, especially those undergoing dialysis treatment and having secondary hyperparathyroidism, have a high risk of bone fracture. The renin-angiotensin system (RAS) is associated with osteoclastic bone resorption. We aimed to examine whether the use of RAS inhibitors reduces the incidence of fracture in hemodialysis patients.

**Methods and Findings:**

This was a multicenter, 3-year, prospective, observational study. From 2008 to 2011, maintenance hemodialysis patients with secondary hyperparathyroidism (N = 3,276) treated with angiotensin converting enzyme inhibitor (ACEI)/angiotensin II receptor blocker (ARB) at baseline were followed for a mean of 2.7 years. The association between the use of ACEI/ARB and hospitalization rate owing to fracture was examined by using Cox regression models. Effect modifications by the severity of secondary hyperparathyroidism (intact parathyroid hormone [iPTH] level), sex, and systolic blood pressure were also examined. The incidence proportion of fracture-related hospitalization was 5.42% throughout the observation period. ACEI/ARB use was associated with a lower rate of fracture-related hospitalization (adjusted hazard ratio, 0.65; 95% confidence interval [CI], 0.45–0.92). This association was not significantly affected by sex (P = 0.56) or systolic blood pressure levels (P = 0.87). The hazard ratios adjusted by iPTH levels were qualitatively different, but not statistically significant (P = 0.11): 0.77 (95% CI, 0.42–1.39), 0.38 (95% CI, 0.20–0.73), 0.59 (95% CI, 0.29–1.21), and 1.29 (95% CI, 0.58–2.42) for the first, second, third and fourth quartiles of iPTH, respectively.

**Conclusions:**

Use of RAS inhibitors is associated with a lower rate of fracture-related hospitalization in hemodialysis patients with secondary hyperparathyroidism.

**Trial Registration:**

ClinicalTrials.gov NCT00995163

## Introduction

Patients with chronic kidney disease (CKD), especially those undergoing dialysis, have poor survival and increased risk of cardiovascular disease (CVD) due to hypertension. To control blood pressure and preserve cardiac function, inhibition of the renin-angiotensin system (RAS) with angiotensin-converting enzyme inhibitors (ACEIs) and angiotensin II receptor blockers (ARBs) is used widely in patients with CKD[1–3].

Mineral and bone metabolic disorders are other common complications of patients with CKD. This disorder contributes to the development of bone fractures, and increases the risk for all-cause and cardiovascular mortality owing to vascular calcification.[4–13] The KDIGO (Kidney Disease: Improving Global Outcomes) and other international/national guidelines [14–16] proposed clinical practices for CKD-associated mineral and bone disorders, by using adequate dialysate, phosphate binders, vitamin D receptor activators, and calcimimetics. For instance, severe secondary hyperparathyroidism, caused by the elevation of circulating parathyroid hormone (PTH), is associated with the incidence of fractures in CKD patients undergoing dialysis treatment[8]. Furthermore, recent improvements in practice patterns have shown that the use of cinacalcet and increased vitamin D receptor activators is associated with lower PTH levels and achievement of target phosphorus levels[17]. Despite these developments in the treatment of mineral metabolic abnormalities, the risk of fracture among patients with CKD undergoing dialysis treatment remains considerably higher than in the general population[18–21]. Therefore, additional therapeutic strategies are required to reduce the incidence of fracture in hemodialysis patients.

Several clinical studies on the general population have suggested that ACEIs decrease the risk of bone fracture[22–24]. The RAS in patients with dialysis treatment is inordinately activated as compared with that in healthy subjects[25], then, there is a possibility that RAS inhibitors are more effective in reducing the incidence of fracture in dialysis patients than in the general population. In this study, we investigated whether the use of RAS inhibitors reduces the incidence of hospitalization owing to bone fractures among hemodialysis patients with secondary hyperparathyroidism. We examined data from the MBD-5D study (The Mineral and Bone Disorder Outcomes Study for Japanese CKD Stage 5D Patients)[26].

## Materials and Methods

### Study design

The MBD-5D study is a multicenter, 3-year, prospective, observational study involving maintenance hemodialysis patients with secondary hyperparathyroidism. Details of the study design have been published elsewhere[26, 27]. Briefly, the MBD-5D began in January 2008 and included 8,229 patients, with a subcohort comprising of randomly selected patients (n = 3,276 [40%]) from whom data on covariates were collected. We used the data from the subcohort in this analysis. The mean observation period was 2.74 years. The adhered to the Declaration of Helsinki. Because this is an observational study with only anonymized data collected from routine practice, informed consent from subjects was not required according to the ethical guidelines for epidemiological research in Japan. The study protocol and the waiver of informed consent were approved by the central ethics committee at Kobe University (no. 754).

### Inclusion and exclusion criteria

Eligible patients comprised all those who were receiving hemodialysis at a participating facility as of January 1, 2008, and who satisfied any of the following inclusion criteria: intact PTH (iPTH) level of ≥180 pg/mL or treatment with intravenous vitamin D receptor activator or oral active vitamin D receptor activator. Patients undergoing hemodialysis for <3 months were excluded.

### Outcomes and exposure

The primary outcome of this study was the time to incidence of hospitalization owing to fracture, regardless of the type of fracture. We examined the period from the initiation of the observation to the occurrence of the initial event. A participant was censored after the initial event. The exposure of interest was ACEI/ARB use at baseline.

### Statistical analyses

To describe patient characteristics, continuous variables are shown as medians (interquartile range: IQR), whereas categorical variables are shown as percentages. For comparisons between the groups with and without ACEI/ARB use, Wilcoxon and chi-square tests were used, respectively.

In the primary analysis, the association between ACEI/ARB use and the incidence of hospitalization owing to fracture was examined. The cumulative incidence of hospitalization owing to fracture between groups with and without ACEI/ARB use was analyzed by using the Kaplan–Meier survival curve and log-rank test. Non-adjusted, case-mix-adjusted, and multivariate-adjusted Cox regression proportional hazard models were used to estimate hazard ratios (HRs) and their 95% confidence intervals (CIs). Deaths (n = 506), losses during the follow-up period (n = 143), renal transplantation (n = 5), changes to peritoneal dialysis (n = 2), and termination of the observation were considered censored data. The proportional hazards assumption was visually checked by using a log negative-log survival plot.

In the case-mix adjusted model, age, sex, duration of dialysis, and causes of end-stage kidney disease were adjusted. In the multivariate-adjusted model, the following covariates were adjusted in addition to those in the case-mix model: body mass index; Kt/V; comorbidity of CVD and/or diabetes mellitus, smoking, history of parathyroidectomy, prescriptions of anticoagulants, vitamin D receptor activators, and phosphate binders, and serum levels of albumin, calcium, phosphorus, PTH, alkaline phosphatase, and blood hemoglobin, in addition to systolic and diastolic blood pressure and the use of antihypertensive drugs (β-blockers, calcium channel blockers, diuretics, and others).

To reinforce the results of the primary analysis, a sensitivity analysis was performed: blood pressure and use of each antihypertensive drug were removed from the covariates of the multivariate-adjusted model to confirm whether the analysis was robust if these blood pressure-related data were intermediates rather than confounders.

In the secondary analysis, effect modifications by some factors clinically relevant to fractures were examined, such as the severity of secondary hyperparathyroidism (i.e., iPTH levels), sex, and systolic blood pressure. The iPTH and systolic blood pressure were categorized into quartiles. We applied Cox regression models in which the main predictor was defined as a categorical combination of one of the factors mentioned above and the use of ACEI/ARB, with the same covariates as those used in the multivariate-adjusted models in the primary analysis. The statistical significance of the effect modification was tested by using a likelihood ratio test.

All statistical analyses were performed with SAS 9.2 (SAS Institute, Cary, NC, USA). A P value of <0.05 was considered significant.

## Results

### Baseline characteristics

The median age of the patients was 63 (IQR, 54–71) years. Women accounted for 38.5%. The median duration of dialysis was 8.3 (IQR, 3.7–14.3) years. Patients with diabetes mellitus and CVD accounted for 31.6% and 60.0%, respectively.

A higher proportion of men than women had diabetic nephropathy as the primary disease among patients using ACEI/ARB compared with those not using ACEI/ARB (31.6% vs. 19.3%, Table 1). Patients using ACEI/ARB had a shorter duration of dialysis, lower iPTH, and alkaline phosphatase levels, and higher systolic blood pressure than those not using ACEI/ARB. Patients using ACEI/ARB also used antihypertensive drugs more frequently, especially calcium antagonists, than those not using ACEI/ARB (68.2% vs. 27.7%). Mortality during the observation period was lower among patients using ACEI/ARB than those not using ACEI/ARB (13.3% vs. 16.8%). Dropouts and loss to follow-up occurred to the same extent among patients using and those not using ACEI/ARB (4.1% and 4.9%, respectively).

**Table 1:** Baseline characteristics of the participants.

			ACEI/ARB		
			Non-user	User	
Variable			n = 1,986	n = 1,290	P-value
*Demographic and clinical characteristics*					
	Age, years		63 (55–71)	62 (54–71)	0.03
	Sex, female, %		41.4	34.0	<0.001
	Body mass index, kg/m^2^		20.9 (19.0–23.4)	20.8 (19.0–23.2)	0.59
	Cause of end-stage kidney disease				<0.001
		Glomerulonephritis, %	48.2	40.0	
		Diabetic nephropathy, %	19.3	34.1	
		Pyelonephritis, %	2.27	1.09	
		Cystic kidney, %	4.88	3.57	
		Nephrosclerosis, %	5.89	5.74	
		Others, %	19.4	18.2	
	Systolic blood pressure, mmHg		148 (131–164)	156 (142–170)	<0.001
	Diastolic blood pressure, mmHg		79 (70–87)	80 (72–90)	<0.001
	Smoking, %		25.1	26.8	0.50
*Co-morbid conditions*					
	Diabetes mellitus, %		27.3	38.2	<0.001
	Cardiovascular disease, %		60.7	58.9	0.32
*Dialysis*					
	Duration of dialysis, years		8.95 (4.08–15.9)	7.05 (3.05–12.0)	<0.001
	Kt/V, single pool		1.42 (1.24–1.61)	1.35 (1.18–1.53)	<0.001
*Laboratory data*					
	Albumin, g/dL		3.8 (3.5–4.0)	3.8 (3.6–4.0)	0.03
	Blood hemoglobin, g/dL		10.6 (9.8–11.3)	10.5 (9.8–11.1)	0.06
	Calcium, mg/dL		9.5 (8.9–10.1)	9.4 (8.8–10.1)	0.35
	Phosphorus, mg/dL		5.4 (4.5–6.3)	5.5 (4.8–6.4)	0.15
	Intact parathyroid hormone, pg/mL		276 (200–409)	252 (189–362)	<0.001
	Alkaline phosphatase, IU/L		259 (199–352)	243 (183–318)	<0.001
*Antihypertensive drugs*					
	β-Blocker, %		4.73	10.6	<0.001
	Calcium channel blocker, %		27.7	68.2	<0.001
	Diuretic, %		10.7	16.0	<0.001
	Others, %		10.2	27.1	<0.001
Anticoagulant therapy, %			5.2	3.5	0.02
Vitamin D receptor activators					0.53
	Intravenous drug, %		48.8	48.6	
	Oral drug, %		28.2	29.6	
	None, %		23.1	21.7	
Phosphate binders					0.40
	Calcium-based drugs, %		43.5	43.7	
	Non-calcium-based drugs, %		19.3	17.1	
	Both, %		22.9	24.0	
	None, %		14.4	15.2	
History of parathyroidectomy, %			7.17	4.76	0.005

For continuous variables, median and interquartile ranges (IQR) are shown.

ACEI: angiotensin converting enzyme inhibitor, ARB: angiotensin II receptor blocker

### Outcomes

In 3,276 patients, there were 178 (5.42%) hospitalizations owing to fractures within the observation period. Of these, 58 hospitalizations were because of hip fractures. Patients using ACEI/ARB had a lower fracture incidence rate (1.48 vs. 2.33 per 100 person-years) and a lower incidence (3.64% vs. 6.60%) throughout the observation period than those not using ACEI/ARB.

### Association between ACEI/ARB use and hospitalization owing to fracture

**Fig 1:**
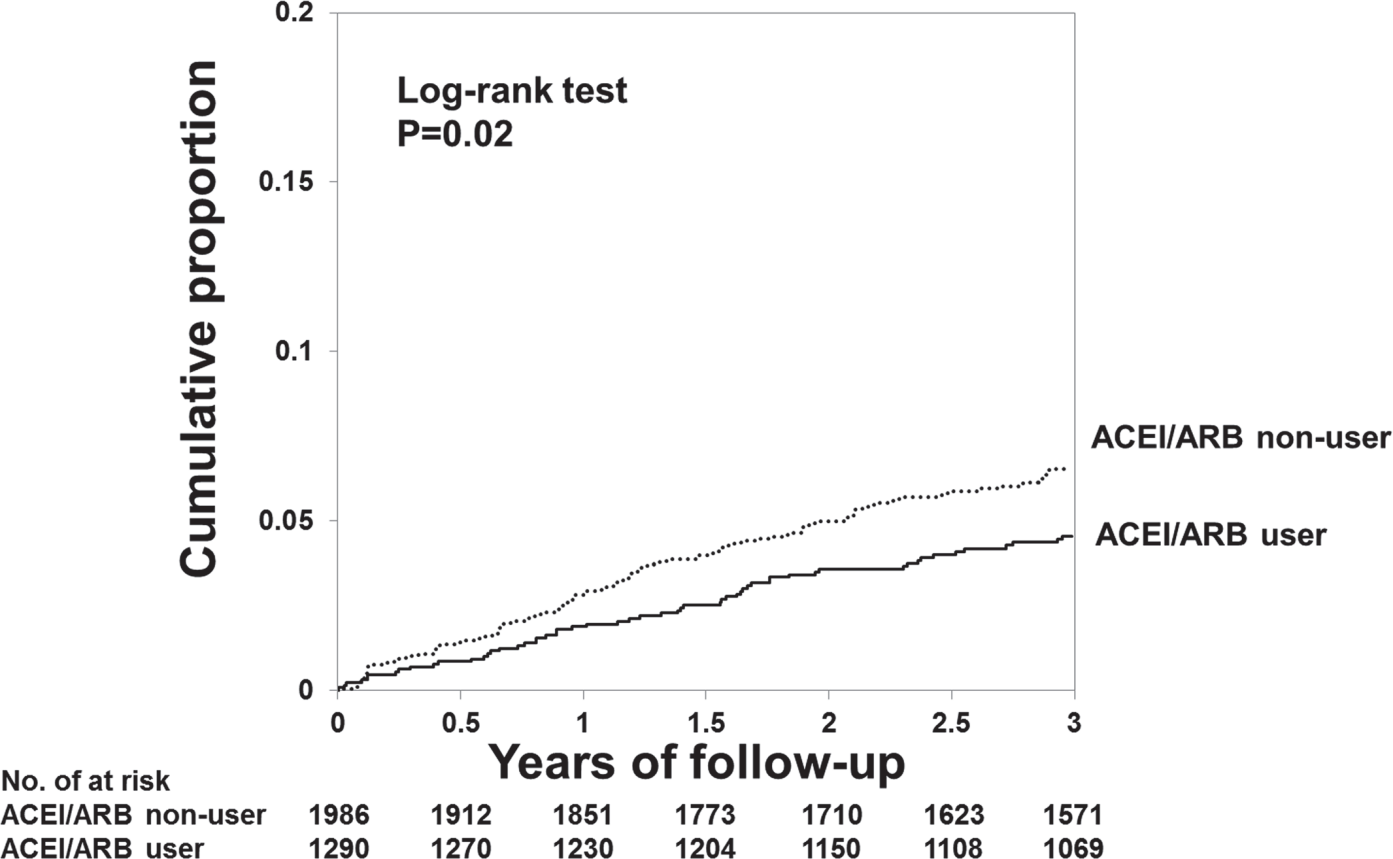
Cumulative proportion of the incidence of hospitalization owing to fractures. ACEI: angiotensin converting enzyme inhibitor, ARB: angiotensin II receptor blocker.

Fig 1 shows the Kaplan-Meier curves of the cumulative proportion of hospitalization owing to fracture. During the 3-year observation period, the cumulative proportions in patients treated with ACEI/ARB and those not treated with ACEI/ARB were 4.55% and 6.54%, respectively (P = 0.02; log-rank test).

**Fig 2:**
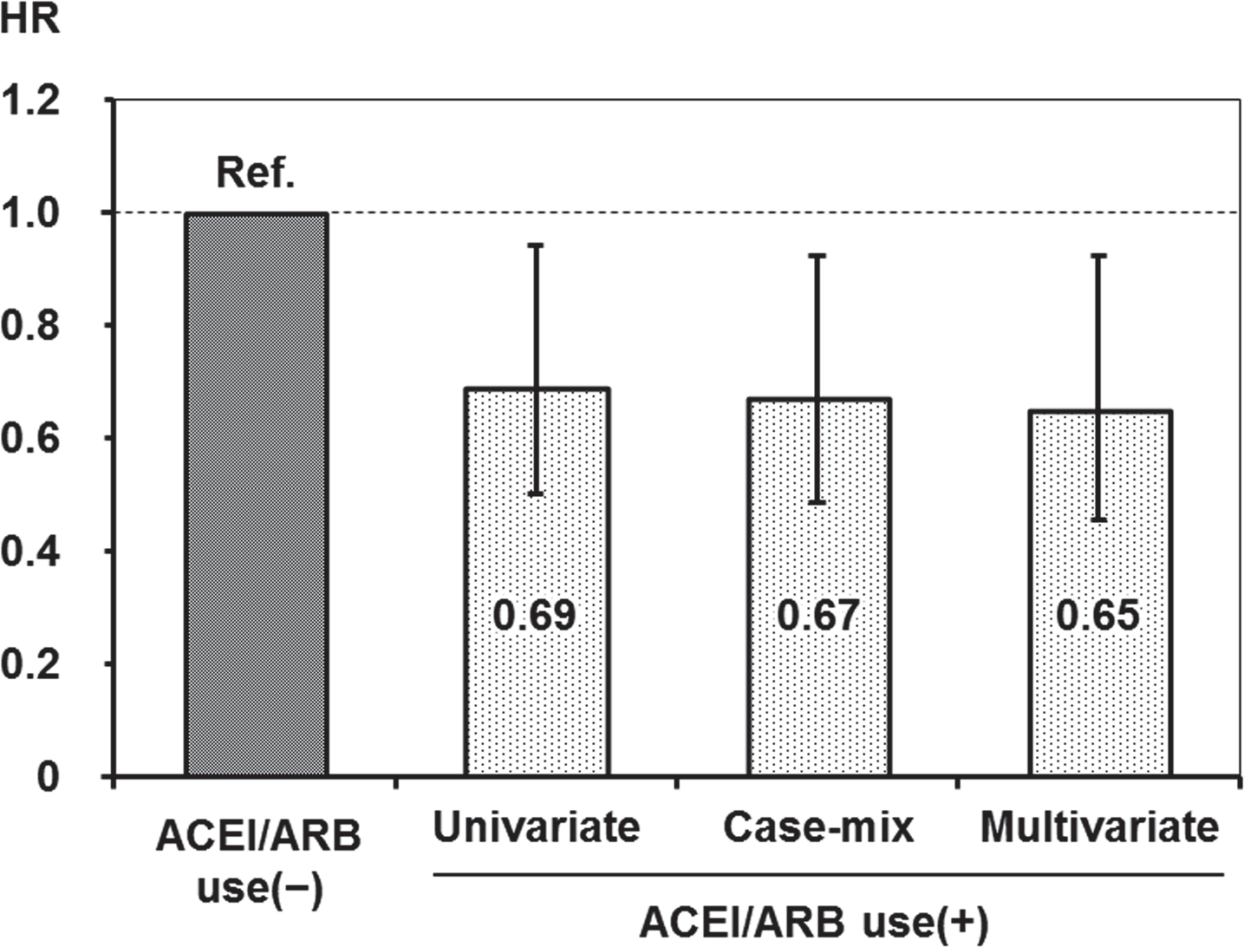
Crude, case-mix-adjusted, and multivariate-adjusted hazard ratios of hospitalization owing to fractures associated with angiotensin-converting enzyme inhibitor/angiotensin II receptor blocker use. The covariates included in each analytical model for adjustment were as follows: case-mix, age, sex, duration of dialysis, and causes of end-stage kidney disease. In the multivariate-adjusted model, in addition to those in the case-mix model, the following covariates were adjusted; body mass index; Kt/V; comorbidity of cardiovascular disease and/or diabetes mellitus; smoking; history of parathyroidectomy; prescriptions of anti-coagulants, vitamin D receptor activators, and phosphate binders; and serum levels of albumin, calcium, phosphorus, intact parathyroid hormone, alkaline phosphatase, and blood hemoglobin, in addition to systolic and diastolic blood pressure and the use of antihypertensive drugs (β-blockers, calcium channel blockers, diuretics, and others). HR: hazard ratio, ACEI: angiotensin-converting enzyme inhibitor, ARB: angiotensin II receptor blocker.

On the basis of the Cox regression analyses, ACEI/ARB use was associated with a lower incidence of hospitalization owing to fracture in non-adjusted (HR, 0.69; 95% CI, 0.50–0.94), case-mix-adjusted (HR, 0.67; 95% CI, 0.49–0.92) and multivariate-adjusted (HR, 0.65; 95% CI, 0.45–0.92) models (Fig 2). Similar results were obtained when blood pressure and use of oral antihypertensive drugs were not included as covariates in the sensitivity analysis (HR: 0.66; 95% CI 0.48–0.91).

### Effect modifications

**Fig 3:**
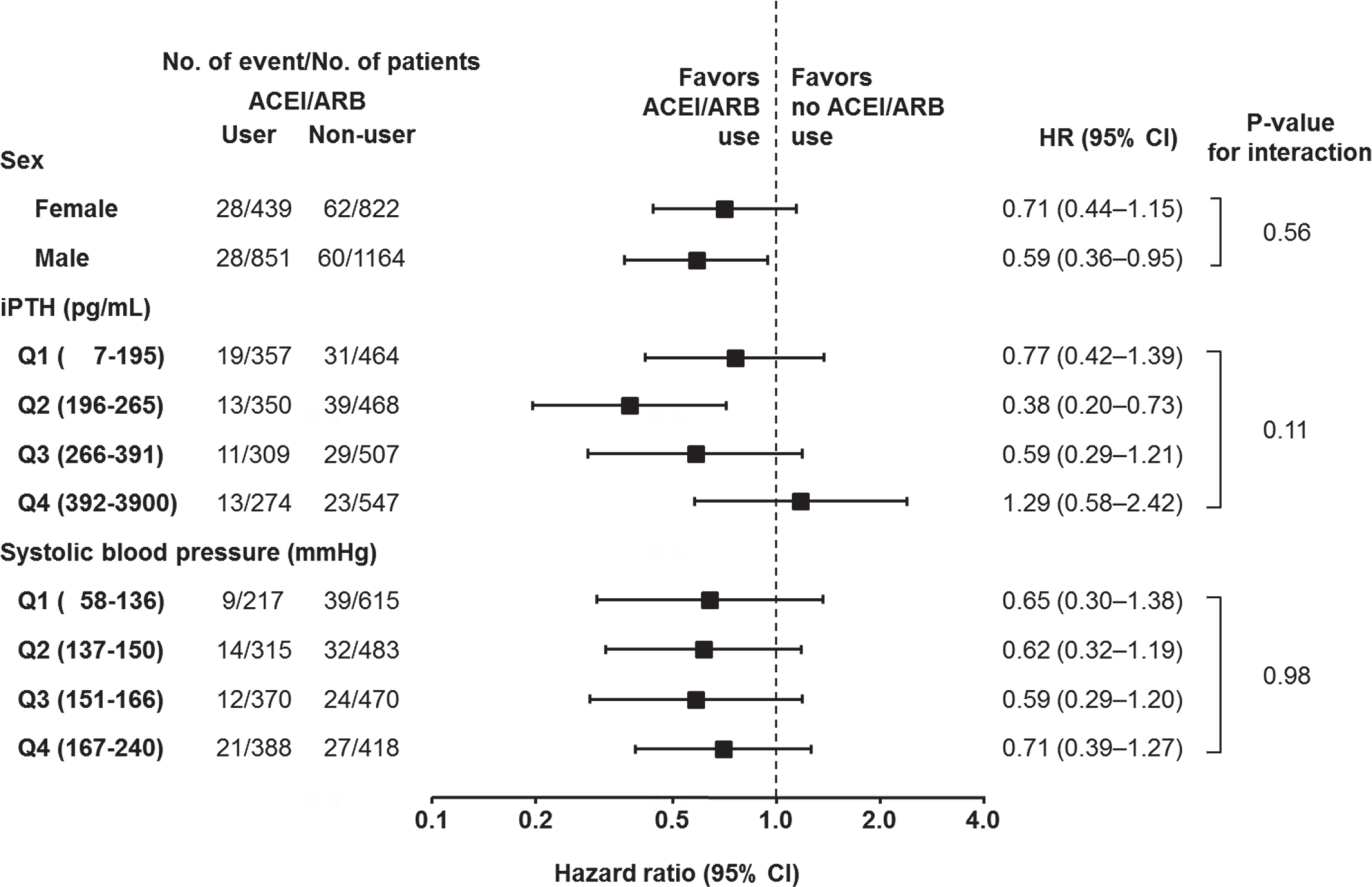
Multivariate-adjusted hazard ratios of hospitalization owing to fractures associated with angiotensin-converting enzyme inhibitor/angiotensin II receptor blocker use considering patients with different levels of parathyroid hormone, sex, or systolic blood pressure. The hazard ratio was obtained from the Cox regression model adjusted for the distribution of age; sex; duration of dialysis; causes of end-stage kidney disease; body mass index; Kt/V; comorbidity of cardiovascular disease or diabetes mellitus; smoking; history of parathyroidectomy; prescriptions of anti-coagulants, vitamin D receptor activators, and phosphate binders; and serum levels of albumin, calcium, phosphorus, parathyroid hormone, alkaline phosphatase, and blood hemoglobin, in addition to systolic and diastolic blood pressure and the use of antihypertensive drugs (β-blockers, calcium channel blockers, diuretics, and others). P values for interactions were obtained from the likelihood ratio test. CI: confidence interval, HR: hazard ratio, iPTH: intact parathyroid hormone, ACEI: angiotensin-converting enzyme inhibitor, ARB: angiotensin II receptor blocker.

The results of the analysis of effect modifications by iPTH levels, sex, and systolic blood pressure for the association between ACEI/ARB use and hospitalization owing to fracture are shown in Fig 3. No significant interactions were observed for sex and systolic blood pressure (P for interaction = 0.56 and 0.87, respectively). The HRs adjusted by iPTH levels were qualitatively different: however the interaction was not statistically significant (P = 0.11): 0.77 (95% CI, 0.42–1.39), 0.38 (95% CI, 0.20–0.73), 0.59 (95% CI, 0.29–1.21), and 1.29 (95% CI 0.58–2.42) for the first (≤less than 195 pg/mL), second (196–265 pg/mL), third (266–391 pg/mL) and fourth (≥392 pg/mL) quartiles of iPTH, respectively.

## Discussion

This study showed that treatment with ACEI/ARB was associated with a lower rate of hospitalization owing to fracture in hemodialysis patients with secondary hyperparathyroidism (Figs 1 and 2). This effect was clear in patients whose serum iPTH levels were controlled at the second (196–265 pg/mL) quartile (Fig 3).

In the general population, use of ACEIs decreases the risk for fracture. A population-based, pharmaco-epidemiological, case-control study showed that the risk of any fracture was reduced by 9% in patients who received ACEIs during the 5-year observation period[24]. The RAS might be involved more strongly in bone metabolism in hemodialysis patients who have inordinately activated RAS as compare to the general population[25]. Indeed, the use of ACEI/ARB led to a 35% reduction in hospitalization owing to fracture in patients undergoing hemodialysis (Figs 1 and 2), which appears to be a much stronger effect than that observed in the general population[24].However, the effect of RAS inhibitors on bone volume and quality has been controversial in clinical and basic researches. A cross-sectional study showed that the use of ACEI is associated with higher bone mineral density in the femoral neck, total neck, and lumbar spine among the elderly population[22], whereas use of ACEI affects bone loss in the elderly[28–30]. In animal models, administration of angiotensin II in rats and nephrectomy in mice decreasebone mass, but not in angiotensin II type 1a receptor-deficient mice[31]. Another study also showed that increase of trabecular number and connectivity with elevation of bone turnover is found in angiotensin II type 1a receptor-deficient mice.[32]. In a murine femur fracture model, perindopril reduced bone mineral density, whereas it improved periosteal callus formation, bone bridging of the fracture gap and torsional stiffness[33]. *In vitro* studies have shown that angiotensin II directly enhances multinuclear osteoclast differentiation from bone marrow-derived mononuclear cells, and induces the expression of receptor activator of nuclear factor-κB ligand in osteoblasts; in contrast, these effects are inhibited by ARBs[31]. Taken together, clinical and basic researches showed a controversial effect of RAS inhibitors on bone volume and quality; however, the current study suggests an obvious relation between use of RAS inhibitors and a lower incidence of fracture. Further study will be needed to understand the change of bone volume and quality with RAS inhibitors in patients with dialysis treatment.

In the analysis of the effect modification by the severity of secondary hyperparathyroidism, the effect of ACEI/ARB on the incidence of fractures was obvious in patients with the second (196–265 pg/mL) quartiles as compare to in those with the first (≤195 pg/mL), third (266–391 pg/mL) and fourth (≥392 pg/mL) quartiles of iPTH (Fig 3). It is possible that because of their low turnover bone state induced by low PTH, treatment with ACEI/ARB had no beneficial effect in those patients. In this condition, low PTH levels induce suppression of osteoclastogenesis, and therefore, the effect of ACEI/ARB would be masked. As mentioned above, patients with high iPTH levels did not show a significant beneficial effect of ACEI/ARB use. Osteoclastogenesis induced by extremely elevated levels of circulating PTH may have overwhelmed the inhibitory effect of ACEI/ARB at the prescribed dosage.

This study has several limitations. The MBD-5D data were obtained from patients at dialysis facilities in Japan. Our cohort had far lower competing risks such as deaths and renal transplantations than those in Western countries, which made it suitable to examine bone fractures as an outcome. However, these results may not be applicable to dialysis patients worldwide. We could not obtain detailed data about fractures such as site, severity, and cause of fracture as well as on bone mineral density. While the events evaluated in this study required hospitalization, we could not obtain data for activities of daily living that may have influenced the outcome. Furthermore, this was an observational study and could not escape from confounding by indication bias, although the sensitivity analyses showed consistent results.

## Conclusions

The use of RAS inhibitors is associated with a lower rate of hospitalization owing to bone fracture in hemodialysis patients with secondary hyperparathyroidism. To the best of our knowledge, this is the first study to report a significant reduction of the fracture risk in patients with severe CKD. RAS intervention may be an appealing preventive therapeutic strategy in addition to conventional treatments available for patients with CKD-associated mineral and bone disorder.

## References

[1] Zannad F, Kessler M, Lehert P, Grunfeld JP, Thuilliez C, Leizorovicz A, et al Prevention of cardiovascular events in end-stage renal disease: results of a randomized trial of fosinopril and implications for future studies. Kidney Int. 2006;70(7):1318–24. Epub 2006/07/28. 10.1038/sj.ki.5001657 . 16871247

[2] Takahashi A, Takase H, Toriyama T, Sugiura T, Kurita Y, Ueda R, et al Candesartan, an angiotensin II type-1 receptor blocker, reduces cardiovascular events in patients on chronic haemodialysis—a randomized study. Nephrol Dial Transplant. 2006;21(9):2507–12. Epub 2006/06/13. 10.1093/ndt/gfl293 . 16766543

[3] Suzuki H, Kanno Y, Sugahara S, Ikeda N, Shoda J, Takenaka T, et al Effect of angiotensin receptor blockers on cardiovascular events in patients undergoing hemodialysis: an open-label randomized controlled trial. Am J Kidney Dis. 2008;52(3):501–6. Epub 2008/07/26. 10.1053/j.ajkd.2008.04.031 . 18653268

[4] Block GA, Klassen PS, Lazarus JM, Ofsthun N, Lowrie EG, Chertow GM. Mineral metabolism, mortality, and morbidity in maintenance hemodialysis. J Am Soc Nephrol. 2004;15(8):2208–18. Epub 2004/07/31. 10.1097/01.ASN.0000133041.27682.A2 . 15284307

[5] Danese MD, Kim J, Doan QV, Dylan M, Griffiths R, Chertow GM. PTH and the risks for hip, vertebral, and pelvic fractures among patients on dialysis. Am J Kidney Dis. 2006;47(1):149–56. Epub 2005/12/27. 10.1053/j.ajkd.2005.09.024 . 16377396

[6] Floege J, Kim J, Ireland E, Chazot C, Drueke T, de Francisco A, et al Serum iPTH, calcium and phosphate, and the risk of mortality in a European haemodialysis population. Nephrol Dial Transplant. 2011;26(6):1948–55. Epub 2010/05/15. 10.1093/ndt/gfq219 20466670 PMC3107766

[7] Goodman WG, Goldin J, Kuizon BD, Yoon C, Gales B, Sider D, et al Coronary-artery calcification in young adults with end-stage renal disease who are undergoing dialysis. N Engl J Med. 2000;342(20):1478–83. Epub 2000/05/18. 10.1056/NEJM200005183422003 . 10816185

[8] Jadoul M, Albert JM, Akiba T, Akizawa T, Arab L, Bragg-Gresham JL, et al Incidence and risk factors for hip or other bone fractures among hemodialysis patients in the Dialysis Outcomes and Practice Patterns Study. Kidney Int. 2006;70(7):1358–66. Epub 2006/08/25. 10.1038/sj.ki.5001754 . 16929251

[9] Kalantar-Zadeh K, Kuwae N, Regidor DL, Kovesdy CP, Kilpatrick RD, Shinaberger CS, et al Survival predictability of time-varying indicators of bone disease in maintenance hemodialysis patients. Kidney Int. 2006;70(4):771–80. Epub 2006/07/06. 10.1038/sj.ki.5001514 . 16820797

[10] Kimata N, Albert JM, Akiba T, Yamazaki S, Kawaguchi T, Fukuhara S, et al Association of mineral metabolism factors with all-cause and cardiovascular mortality in hemodialysis patients: the Japan dialysis outcomes and practice patterns study. Hemodial Int. 2007;11(3):340–8. Epub 2007/06/20. 10.1111/j.1542-4758.2007.00190.x . 17576300

[11] Nakai S, Akiba T, Kazama J, Yokoyama K, Fukagawa M, Tominaga Y, et al Effects of serum calcium, phosphorous, and intact parathyroid hormone levels on survival in chronic hemodialysis patients in Japan. Therapeutic apheresis and dialysis: official peer-reviewed journal of the International Society for Apheresis, the Japanese Society for Apheresis, the Japanese Society for Dialysis Therapy. 2008;12(1):49–54. Epub 2008/02/09. 10.1111/j.1744-9987.2007.00540.x . 18257812

[12] Raggi P, Boulay A, Chasan-Taber S, Amin N, Dillon M, Burke SK, et al Cardiac calcification in adult hemodialysis patients. A link between end-stage renal disease and cardiovascular disease? J Am Coll Cardiol. 2002;39(4):695–701. Epub 2002/02/19. . 11849871 10.1016/s0735-1097(01)01781-8

[13] Tentori F, Blayney MJ, Albert JM, Gillespie BW, Kerr PG, Bommer J, et al Mortality risk for dialysis patients with different levels of serum calcium, phosphorus, and PTH: the Dialysis Outcomes and Practice Patterns Study (DOPPS). Am J Kidney Dis. 2008;52(3):519–30. Epub 2008/06/03. 10.1053/j.ajkd.2008.03.020 . 18514987

[14] K/DOQI clinical practice guidelines for bone metabolism and disease in chronic kidney disease. Am J Kidney Dis. 2003;42(4 Suppl 3):S1–201. Epub 2003/10/02. . 14520607

[15] KDIGO clinical practice guideline for the diagnosis, evaluation, prevention, and treatment of Chronic Kidney Disease-Mineral and Bone Disorder (CKD-MBD). Kidney Int Suppl. 2009;(113):S1–130. Epub 2009/08/01. 10.1038/ki.2009.188 . 19644521

[16] Fukagawa M, Yokoyama K, Koiwa F, Taniguchi M, Shoji T, Kazama JJ, et al Clinical practice guideline for the management of chronic kidney disease-mineral and bone disorder. Therapeutic apheresis and dialysis: official peer-reviewed journal of the International Society for Apheresis, the Japanese Society for Apheresis, the Japanese Society for Dialysis Therapy. 2013;17(3):247–88. Epub 2013/06/06. 10.1111/1744-9987.12058 . 23735142

[17] Fukagawa M, Fukuma S, Onishi Y, Yamaguchi T, Hasegawa T, Akizawa T, et al Prescription patterns and mineral metabolism abnormalities in the cinacalcet era: results from the MBD-5D study. Clin J Am Soc Nephrol. 2012;7(9):1473–80. Epub 2012/07/24. 10.2215/CJN.13081211 22822017 PMC3430956

[18] Alem AM, Sherrard DJ, Gillen DL, Weiss NS, Beresford SA, Heckbert SR, et al Increased risk of hip fracture among patients with end-stage renal disease. Kidney Int. 2000;58(1):396–9. Epub 2000/07/08. 10.1046/j.1523-1755.2000.00178.x . 10886587

[19] Coco M, Rush H. Increased incidence of hip fractures in dialysis patients with low serum parathyroid hormone. Am J Kidney Dis. 2000;36(6):1115–21. Epub 2000/11/30. 10.1053/ajkd.2000.19812 . 11096034

[20] Ensrud KE, Lui LY, Taylor BC, Ishani A, Shlipak MG, Stone KL, et al Renal function and risk of hip and vertebral fractures in older women. Arch Intern Med. 2007;167(2):133–9. Epub 2007/01/24. 10.1001/archinte.167.2.133 . 17242313

[21] Wakasugi M, Kazama JJ, Taniguchi M, Wada A, Iseki K, Tsubakihara Y, et al Increased risk of hip fracture among Japanese hemodialysis patients. J Bone Miner Metab. 2013;31(3):315–21. Epub 2013/01/08. 10.1007/s00774-012-0411-z . 23292163

[22] Lynn H, Kwok T, Wong SY, Woo J, Leung PC. Angiotensin converting enzyme inhibitor use is associated with higher bone mineral density in elderly Chinese. Bone. 2006;38(4):584–8. Epub 2005/11/01. 10.1016/j.bone.2005.09.011 . 16257280

[23] Perez-Castrillon JL, Silva J, Justo I, Sanz A, Martin-Luquero M, Igea R, et al Effect of quinapril, quinapril-hydrochlorothiazide, and enalapril on the bone mass of hypertensive subjects: relationship with angiotensin converting enzyme polymorphisms. Am J Hypertens. 2003;16(6):453–9. Epub 2003/06/12. . 12799093 10.1016/s0895-7061(03)00845-8

[24] Rejnmark L, Vestergaard P, Mosekilde L. Treatment with beta-blockers, ACE inhibitors, and calcium-channel blockers is associated with a reduced fracture risk: a nationwide case-control study. J Hypertens. 2006;24(3):581–9. Epub 2006/02/10. 10.1097/01.hjh.0000203845.26690.cb . 16467662

[25] Kovarik JJ, Antlanger M, Domenig O, Kaltenecker CC, Hecking M, Haidinger M, et al. Molecular regulation of the renin-angiotensin system in haemodialysis patients. Nephrol Dial Transplant. 2014. Epub 2014/08/12. 10.1093/ndt/gfu265 . 25107336

[26] Fukuhara S, Akizawa T, Fukagawa M, Onishi Y, Yamaguchi T, Hasegawa T, et al Mineral and bone disorders outcomes study for Japanese chronic kidney disease stage 5D patients: rationale and study design. Therapeutic apheresis and dialysis: official peer-reviewed journal of the International Society for Apheresis, the Japanese Society for Apheresis, the Japanese Society for Dialysis Therapy. 2011;15(2):169–75. Epub 2011/03/24. 10.1111/j.1744-9987.2010.00906.x . 21426510

[27] Fukagawa M, Komaba H, Onishi Y, Fukuhara S, Akizawa T, Kurokawa K. Mineral metabolism management in hemodialysis patients with secondary hyperparathyroidism in Japan: baseline data from the MBD-5D. Am J Nephrol. 2011;33(5):427–37. Epub 2011/04/22. 10.1159/000327654 . 21508631

[28] Kwok T, Leung J, Zhang YF, Bauer D, Ensrud KE, Barrett-Connor E, et al Does the use of ACE inhibitors or angiotensin receptor blockers affect bone loss in older men? Osteoporos Int. 2012;23(8):2159–67. Epub 2011/11/15. 10.1007/s00198-011-1831-7 22080379 PMC3772278

[29] Zhang YF, Qin L, Leung PC, Kwok TC. The effect of angiotensin-converting enzyme inhibitor use on bone loss in elderly Chinese. J Bone Miner Metab. 2012;30(6):666–73. Epub 2012/06/30. 10.1007/s00774-012-0363-3 . 22743851

[30] Masunari N, Fujiwara S, Nakata Y, Furukawa K, Kasagi F. Effect of angiotensin converting enzyme inhibitor and benzodiazepine intake on bone loss in older Japanese. Hiroshima J Med Sci. 2008;57(1):17–25. Epub 2008/06/27. . 18578363

[31] Shimizu H, Nakagami H, Osako MK, Hanayama R, Kunugiza Y, Kizawa T, et al Angiotensin II accelerates osteoporosis by activating osteoclasts. FASEB J. 2008;22(7):2465–75. Epub 2008/02/08. 10.1096/fj.07-098954 . 18256306

[32] Kaneko K, Ito M, Fumoto T, Fukuhara R, Ishida J, Fukamizu A, et al Physiological function of the angiotensin AT1a receptor in bone remodeling. Journal of bone and mineral research: the official journal of the American Society for Bone and Mineral Research. 2011;26(12):2959–66. Epub 2011/09/03. 10.1002/jbmr.501 . 21887703

[33] Garcia P, Schwenzer S, Slotta JE, Scheuer C, Tami AE, Holstein JH, et al Inhibition of angiotensin-converting enzyme stimulates fracture healing and periosteal callus formation—role of a local renin-angiotensin system. Br J Pharmacol. 2010;159(8):1672–80. Epub 2010/03/18. 10.1111/j.1476-5381.2010.00651.x 20233225 PMC2925490

